# Older Adults’ Worry about COVID-19: Associations with Experiences of COVID-19 Among Social Connections

**DOI:** 10.1093/geroni/igab046.2749

**Published:** 2021-12-17

**Authors:** Amanda Leggett, Hyun Jung Koo, Lindsay Kobayashi, Jessica Finlay, Hannah Lee, Elaina Baker

**Affiliations:** 1 The University of Michigan, Ypsilanti, Michigan, United States; 2 University of Michigan, Ann Arbor, Michigan, United States

## Abstract

The COVID-19 pandemic has challenged the physical and mental health of older adults, yet it is unknown how much older adults worry about their own exposure. As older adults are at increased risk for severe complications from COVID-19, understanding patterns of worry may inform public health guidelines and interventions for this age group. We investigated older adults’ worry about COVID-19 in the early months of the pandemic and associations with familial/friend’s diagnosis or disease symptoms. Data comes from the baseline (April/May 2020), one-month, and two-month follow-up surveys from the COVID-19 Coping Study, a national longitudinal cohort study of US adults aged ≥55. We used linear regression models to investigate the association between self-reported familial/friend diagnosis or symptoms with pandemic worry, accounting for demographic factors and individual diagnosis or experience of COVID-19 symptoms. Participants (Baseline=4379, 1 month= 2553, 2 month=2682) were 67 years old on average, 72% were female, 5.7% were non-White, and 80.5% had a college degree. At baseline, 26.6% of participants had friends or family who had been diagnosed or experienced symptoms of COVID-19. Having friends or family diagnosed or with symptoms of COVID-19 (B=0.08, SE=0.04, p<.05), being female (B=0.42, SE=0.03, p<.001), and having higher educational attainment (B=0.06, SE=0.02, p<.001) were significantly associated with greater worry about COVID-19. These associations were consistent over 3 months. Understanding if worry about the pandemic correlates with following public health guidelines is a key next step so intervention strategies can prioritize older adults and their social networks.

